# Integrating sarcopenia into ICU-acquired weakness risk stratification: a machine learning–based prediction model for critical care

**DOI:** 10.3389/fnut.2026.1823112

**Published:** 2026-05-14

**Authors:** Peng Zheng, Xinwei Liu, Chunxia Zhang, Wei Zhang, Sheng Yang, Yong Chen, Yong Ye

**Affiliations:** Department of Intensive Care Unit, Clinical Oncology School of Fujian Medical University, Fujian Cancer Hospital, NHC Key Laboratory of Cancer Metabolism, Fuzhou, Fujian, China

**Keywords:** ICU-acquired weakness, machine learning, risk stratification, sarcopenia, XGBoost

## Abstract

**Background:**

Sarcopenia is closely associated with weakness in elderly and various chronic disease populations. However, its specific role as a predisposing factor for ICU-acquired weakness (ICU-AW) in critically ill patients remains unclear. This study aimed to investigate whether sarcopenia is a predictor of ICU-AW and to develop a machine learning-based prediction model integrating sarcopenia for risk stratification.

**Methods:**

A retrospective analysis was conducted on data from a prospectively maintained database. A total of 1,324 critically ill patients were enrolled and randomly divided into a training set (*n* = 927) and a validation set (*n* = 397) in a 7:3 ratio. Sarcopenia was assessed by measuring the skeletal muscle area at the third lumbar vertebra (L3) level using computed tomography. Least absolute shrinkage and selection operator (LASSO) regression and the Boruta algorithm were employed to identify key predictors. 10 machine learning models were developed and their predictive performance was compared. SHAP (SHapley Additive exPlanations) analysis was used to quantify the feature importance of each predictor in the optimal model.

**Results:**

Six predictors were identified by intersecting the results from LASSO regression and the Boruta algorithm in the training set: age, APACHE II score, sarcopenia, sepsis, mechanical ventilation, and lactic acid. Among the 10 machine learning models developed, the XGBoost model exhibited the best overall predictive performance in the training set, achieving an area under the curve (AUC) of 0.838 (95% confidence interval [CI]: 0.807–0.868) and the lowest Brier score of 0.137. Decision curve analysis and clinical impact curves confirmed its stable clinical predictive value. SHAP analysis revealed that sarcopenia ranked as the third most important predictor, following APACHE II and age. In the validation set, the XGBoost model maintained excellent discriminative ability and predictive performance, with an AUC of 0.843 (95% CI: 0.804–0.882) and good calibration, demonstrating a positive net benefit across a threshold probability range of 9%−92%.

**Conclusion:**

Sarcopenia is an important predictor of ICU-AW in critically ill patients. Integrating sarcopenia into the XGBoost model effectively identifies high-risk patients, providing a valuable tool for early risk stratification in intensive care settings.

## Introduction

Advances in critical care medicine have substantially reduced in-hospital mortality among critically ill patients. However, a growing body of evidence indicates that survivors often face significant long-term morbidity and functional impairments following hospital discharge (1, 2). Among these complications, intensive care unit–acquired weakness (ICU-AW) has emerged as a prevalent and debilitating condition. ICU-AW is characterized by generalized, symmetric limb weakness, predominantly affecting proximal muscles of the shoulders and hips with relative sparing of distal muscles, while typically sparing facial and ocular muscles. Its reported incidence ranges from 30 to 50% in general ICU populations and exceeds 75% in patients with sepsis (3–5). Beyond prolonging mechanical ventilation and ICU stay, ICU-AW is strongly associated with increased long-term mortality, delayed physical recovery, reduced health-related quality of life, and substantial healthcare resource utilization (6–8).

To date, no effective pharmacological therapies exist for ICU-AW, and management remains primarily supportive. Prevention is therefore the cornerstone of clinical management, focusing on early rehabilitation and nutritional therapy. Early mobilization protocols—including passive/active range-of-motion exercises, bed cycling, gradual ambulation, and neuromuscular electrical stimulation when appropriate—mitigate muscle wasting, while individualized nutritional support (early enteral nutrition and optimized protein delivery) helps preserve muscle mass (9–14). Guidelines from the United States, along with a growing body of research evidence, confirm the effectiveness of these interventions (15–17). Preventing ICU-AW not only shortens mechanical ventilation duration and reduces ICU stay but also lowers long-term mortality and improves post-discharge physical function and quality of life (18). However, such measures are resource-intensive, requiring specialized personnel, equipment, and time, which are not universally available in all ICUs. Hence, early identification of high-risk patients is essential to efficiently allocate limited resources, delivering timely preventive interventions to those most likely to develop ICU-AW while avoiding unnecessary burden on low-risk patients.

Sarcopenia is a progressive and generalized skeletal muscle disorder characterized by the progressive loss of muscle mass, strength, and function, which is associated with adverse health outcomes such as impaired mobility, increased risk of falls and fractures, prolonged hospitalization, and elevated mortality (19–21). Sarcopenia and frailty are closely interrelated conditions, sharing significant overlap in pathophysiological mechanisms, risk factors, and adverse health outcomes, particularly evident in older adults and across various chronic disease populations such as those with cirrhosis and chronic kidney disease (22–25). Moreover, sarcopenia has been identified as an important predictor of frailty in patients with acute coronary syndrome (26). Critically ill patients admitted to the ICU frequently experience profound metabolic stress, systemic inflammation, prolonged immobilization, and insufficient nutritional intake, all of which rapidly accelerate skeletal muscle breakdown, reduce muscle protein synthesis, and impair mitochondrial function, pathological processes that directly precipitate or worsen pre-existing sarcopenia (27). This ICU-related deterioration in muscle quantity and quality creates a state of diminished muscular reserve and heightened catabolic vulnerability, which in turn acts as a critical biological substrate that predisposes patients to the development of ICU-AW (28). Notably, despite the well-established association between sarcopenia and frailty across diverse disease populations, the specific relationship between ICU-AW and sarcopenia remains largely unexplored.

Therefore, to address this unmet clinical need, this study aimed to investigate whether sarcopenia is a predictor of ICU-AW. Furthermore, we sought to develop and validate a machine learning-based prediction model that integrates sarcopenia for early risk stratification in critically ill patients, thereby enabling more precise allocation of preventive resources in the intensive care unit.

## Materials and methods

### Data and participants

**Figure 1:**
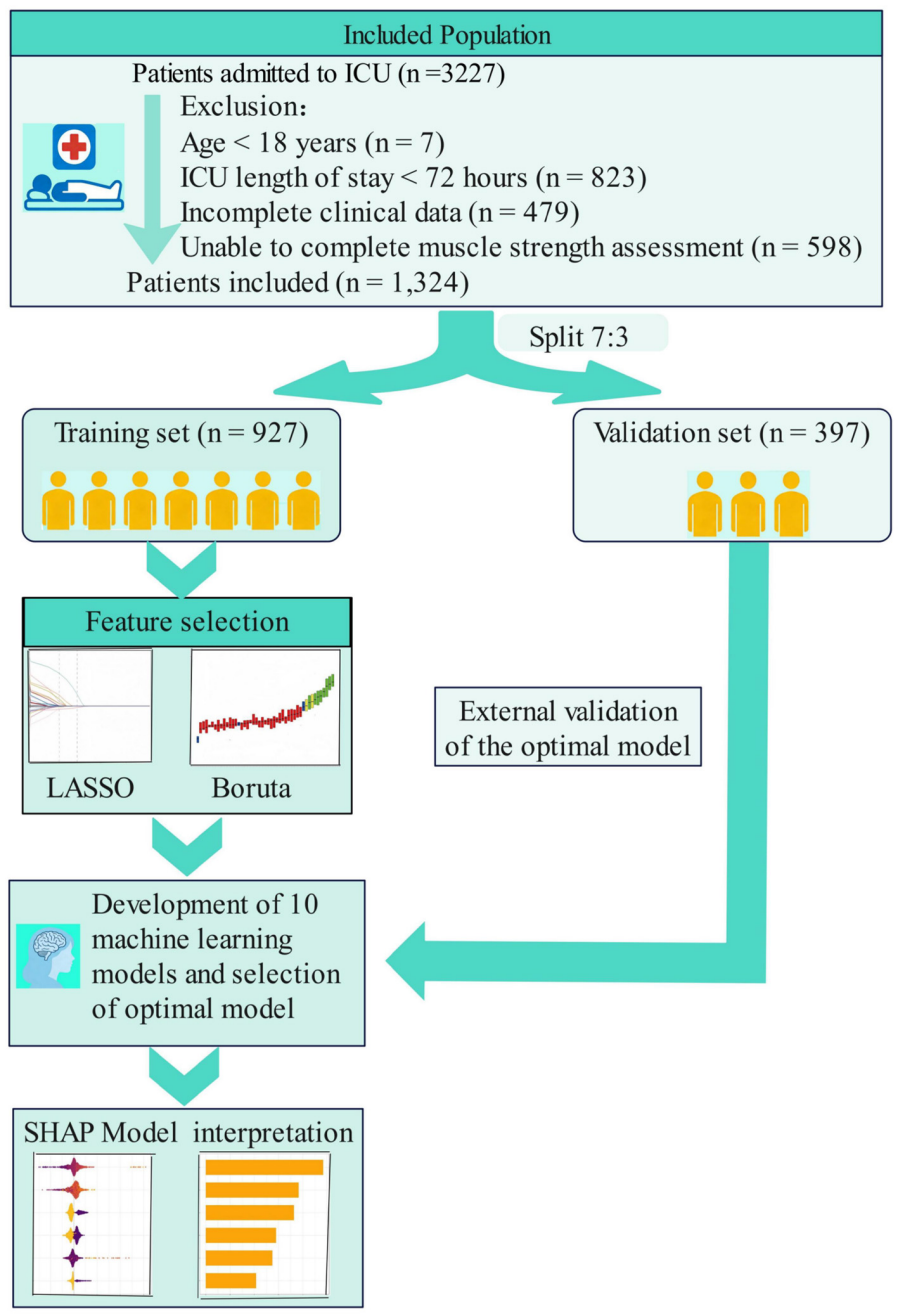
Flow diagram of the study.

This study conducted a retrospective analysis of a prospectively maintained database of patients admitted to the ICU of Fujian Cancer Hospital between January 2022 and July 2025. Inclusion criteria were: (1) age ≥ 18 years; (2) ICU length of stay ≥ 72 h; (3) complete clinical data, including availability of abdominal computed tomography (CT) images within 48 h before or after ICU admission for sarcopenia assessment. Exclusion criteria were: (1) central nervous system diseases (e.g., stroke) or neuromuscular diseases (e.g., Guillain-Barré syndrome, myasthenia gravis, amyotrophic lateral sclerosis); (2) inability to complete muscle strength assessment due to severe cognitive impairment, end-stage diseases precluding cooperation, death during ICU treatment, or withdrawal of life-sustaining therapy. Among the 479 patients excluded due to incomplete data, 289 were excluded specifically because abdominal CT within 48 h of ICU admission was not available. A total of 1,324 patients were enrolled and randomly divided into a training set (*n* = 927) and a validation set (*n* = 397) in a 7:3 ratio. The study flow chart is presented in Figure 1.

### Candidate variable selection

Candidate variables were selected based on clinical rationale, previous literature, and data availability (29–32). Specifically, we included demographic characteristics, comorbidities, disease severity scores, ICU-related exposures, admission laboratory tests, and nutritional support methods as candidate predictors. All candidate predictors were strictly collected at ICU admission or within 48 h of admission.

### Measurements

Sarcopenia was assessed using abdominal CT images acquired within 48 h before or after ICU admission. The cross-sectional area of skeletal muscle at the third lumbar vertebra (L3) level was measured using SliceOmatic software (Supplementary Figure 1). The skeletal muscle area (SMA) was normalized by height squared to calculate the skeletal muscle index (SMI, cm^2^/m^2^). Sarcopenia was defined according to previously established cutoff values for the Asian population: SMI < 34.9 cm^2^/m^2^ in female patients and < 40.8 cm^2^/m^2^ in male patients (33).

ICU-AW was diagnosed using the Medical Research Council (MRC) sum score. Assessments were performed only when patients were awake and able to follow at least three of five simple verbal commands: “open/close your eyes”, “look at me”, “open your mouth and stick out your tongue”, “nod your head”, and “raise your eyebrows when I count to five”. For those staying < 7 days, a single assessment was performed at ICU discharge. For those with an ICU stay ≥7 days, muscle strength was assessed starting on day 7, with evaluations repeated every other day until discharge plus an additional assessment on the day of discharge. The MRC sum score evaluates muscle strength in 12 muscle groups (shoulder abduction, elbow flexion, wrist extension, hip flexion, knee extension, and ankle dorsiflexion bilaterally), with each muscle group scored from 0 (no contraction) to 5 (normal power), yielding a total score ranging from 0 to 60. A score of 60 indicates normal muscle strength, while a score below 48 was defined as ICU-AW, according to established diagnostic criteria (30, 34, 35).

### Statistical analysis

All statistical analyses were performed using R software (version 4.5.2) and SPSS (version 25.0). Continuous variables were presented as mean ± standard deviation (SD) and compared using the independent *t*-test for normally distributed data, or as median with interquartile range (IQR) and compared using the Mann–Whitney *U*-test for non-normally distributed data, based on the results of normality tests. Categorical variables were expressed as numbers (percentages) and compared using the chi-square test.

Before model development, missing values were handled as follows: continuous variables with a missing rate < 10% were imputed using the median, and categorical variables with a missing rate < 10% were imputed using the mode; variables with a missing rate ≥10% were excluded from the candidate pool. To ensure comparability across features, all continuous variables were standardized using z-score normalization (mean = 0, standard deviation = 1) prior to model training. In the training set, feature selection was conducted using two complementary methods: least absolute shrinkage and selection operator (LASSO) regression and the Boruta algorithm. Multicollinearity among the selected predictors was assessed using Pearson correlation coefficients, with *|r|* < 0.4 indicating no significant collinearity.

Ten machine learning algorithms were developed and compared in the training set, including: K-nearest neighbors (KNN), extreme gradient boosting (XGBoost), logistic regression (LR), support vector machine (SVM), random forest (RF), Gaussian naive Bayes (GNB), light gradient boosting machine (LGBM), categorical boosting (CatBoost), decision tree (DT), and multilayer perceptron (MLP). To address class imbalance, class weights were set inversely proportional to class frequencies during model training. To optimize hyperparameters and prevent overfitting, we performed 10-fold cross-validation on the training set for each algorithm. Hyperparameter tuning was conducted using grid search combined with cross-validation; the final hyperparameters for each model are provided in Supplementary Table 1. Model performance was then evaluated using the same cross-validation scheme, reporting the mean area under the receiver operating characteristic curve (AUC) with 95% confidence interval (CI), calibration curves, decision curve analysis, clinical impact curves, accuracy, sensitivity, specificity, positive predictive value (PPV), negative predictive value (NPV), and F1 score. The optimal model was selected based on its comprehensive performance in the training set and subsequently validated in the validation set. A two-tailed *P*-value < 0.05 was considered statistically significant.

## Results

### Comparison of baseline characteristics between training and validation sets

The incidence of ICU-AW was 295/927 (31.8%) in the training set and 138/397 (34.8%) in the validation set. As shown in Table 1, the baseline characteristics between the two cohorts were comparable with no statistically significant differences (*p* > 0.05) for all variables except marital history (*p* = 0.019). However, marital history was not selected as a predictor in our final model nor is it a known risk factor for ICU-AW; therefore, this isolated difference is unlikely to affect model performance.

**Table 1:** Baseline characteristics of the training and validation sets.

Characteristic	Training set (*n* = 927)	Validation set (*n* = 397)	*P* value
Age, mean (SD), year	61.8 ± 11.1	60.8 ± 10.6	0.156
Gender			
Male	500 (53.9)	192 (48.4)	0.063
Female	427 (46.1)	205 (51.6)	
BMI, mean (SD), kg/m^2^	22.1 ± 2.3	22.2 ± 2.3	0.231
History of alcoholism			
Yes	142 (15.3)	53 (13.4)	0.354
No	785 (84.7)	344 (86.6)	
History of smoking			
Yes	187 (20.2)	72 (18.1)	0.392
No	740 (79.8)	325 (81.9)	
History of marital			
Yes	831 (89.6)	338 (85.1)	0.019
No	96 (10.4)	59 (14.9)	
16-7.4,-13.5242ptAPACHE II at ICU admission, mean (SD)	19.9 ± 5.9	19.6 ± 5.8	0.294
Diabetes			
Yes	257 (27.7)	102 (25.7)	0.446
No	670 (72.3)	295 (74.3)	
Hypertension			
Yes	274 (29.6)	130 (32.7)	0.248
No	653 (70.4)	267 (67.3)	
Sarcopenia			
Yes	288 (31.1)	107 (27.0)	0.134
No	639 (68.9)	290 (73.0)	
Heart disease			
Yes	158 (17.0)	82 (20.7)	0.118
No	769 (83.0)	315 (79.3)	
Sepsis			
Yes	223 (24.1)	108 (27.2)	0.225
No	704 (75.9)	289 (72.8)	
DIC			
Yes	182 (19.6)	86 (21.7)	0.400
No	745 (80.4)	311 (78.3)	
Acute renal injury			
Yes	159 (17.2)	79 (19.9)	0.233
No	768 (82.8)	318 (80.1)	
Shock			
Yes	214 (23.1)	76 (19.1)	0.112
No	713 (76.9)	321 (80.9)	
Surgery			
Yes	258 (27.8)	116 (29.2)	0.607
No	669 (72.2)	281 (70.8)	
MV			
Yes	501 (54.0)	213 (53.7)	0.895
No	426 (46.0)	184 (46.3)	
CRRT			
Yes	95 (10.2)	37 (9.3)	0.605
No	832 (89.8)	360 (90.7)	
Corticosteroids			
Yes	198 (21.4)	79 (19.9)	0.550
No	729 (78.6)	318 (80.1)	
Sedative use			
Yes	292 (31.5)	112 (28.2)	0.234
No	635 (68.5)	285 (71.8)	
Opioid use			
Yes	250 (27.0)	97 (24.4)	0.336
No	677 (73.0)	300 (75.6)	
NMBAs			
Yes	197 (21.3)	70 (17.6)	0.133
No	730 (78.7)	327 (82.4)	
Vasoactive medications			
Yes	263 (28.4)	100 (25.2)	0.234
No	664 (71.6)	297 (74.8)	
Protective constraints			
Yes	627 (67.6)	263 (66.2)	0.621
No	300 (32.4)	134 (33.8)	
ICU Admission Labs			
WBC, median (IQR), 10^9^/L	11.9 (8.6, 16.8)	12.5 (8.8, 18.5)	0.053
PLT, median (IQR), 10^9^/L	181.0 (128.0, 250.5)	181.0 (134.0, 256.0)	0.662
Hemoglobin, median (IQR), g/L	124.0 (106.0, 138.0)	127.0 (109.0, 138.0)	0.166
Creatinine, median (IQR), μmol/L	97.2 (70.7, 159.1)	97.2 (70.7, 176.8)	0.454
BUN, median (IQR), mmol/L	7.5 (5.0, 13.2)	7.9 (5.4, 15.4)	0.296
AST, median (IQR), U/L	17.0 (12.0, 27.0)	18.0 (11.0, 25.0)	0.818
ALT, median (IQR), U/L	22.0 (18.0, 30.0)	22.0 (17.0, 28.0)	0.117
T-BIL, median (IQR), μmol/L	9.0 (5.0, 19.0)	11.0 (5.0, 20.0)	0.055
Albumin, median (IQR), g/dl	2.9 (2.5, 3.5)	3.0 (2.6, 3.5)	0.154
Globulin, median (IQR), g/dl	2.5 (2.1, 3.0)	2.6 (2.1, 3.2)	0.130
Triglycerides, median (IQR), mmol/L	1.4 (0.9, 2.3)	1.45 (0.9, 2.2)	0.802
Cholesterol, median (IQR), mmol/L	5.0 (4.3, 5.8)	5.1 (4.4, 5.8)	0.370
CRP, median (IQR), mg/L	107.9 (48.1, 189.4)	111.5 (50.9, 192.6)	0.402
Glucose, median (IQR), mmol/L	13.2 (10.6, 17.1)	13.6 (11.1, 17.0)	0.251
Calcium, median (IQR), mmol/L	2.1 (1.9, 2.2)	2.1 (2.0, 2.3)	0.559
Sodium, median (IQR), mmol/L	137.0 (133.0, 141.0)	137.0 (133.0, 141.0)	0.466
Potassium, median (IQR), mmol/L	4.0 (3.5, 4.5)	3.9 (3.4, 4.5)	0.207
Chloride, median (IQR), mmol/L	104.0 (99.0, 108.0)	104.0 (101.0, 109.0)	0.061
16-7.4,-13.5242ptLactic acid, median (IQR), mmol/L	1.90 (1.3, 3.1)	2.00 (1.2, 3.2)	0.561
Nutritional support methods			
Extraintestinal + Intraintestinal	436 (47.0)	201 (50.6)	0.230
Total parenteral nutrition	491 (53.0)	196 (49.4)	

ICU, intensive care unit; ICU-AW, ICU-acquired weakness; BMI, body mass index; APACHE II, acute physiology and chronic health evaluation II; DIC, disseminated intravascular coagulation; MV, mechanical ventilation; CRRT, continuous renal replacement therapy; NMBAs, neuromuscular blocking agents; Labs, laboratory tests; WBC, white blood cell; PLT, platelet; CRP, C-reactive protein; BUN, blood urea nitrogen; AST, aspartate aminotransferase; ALT, alanine aminotransferase; T-BIL, total bilirubin.

### Screening of influencing factors for ICU-AW in the training set

**Figure 2:**
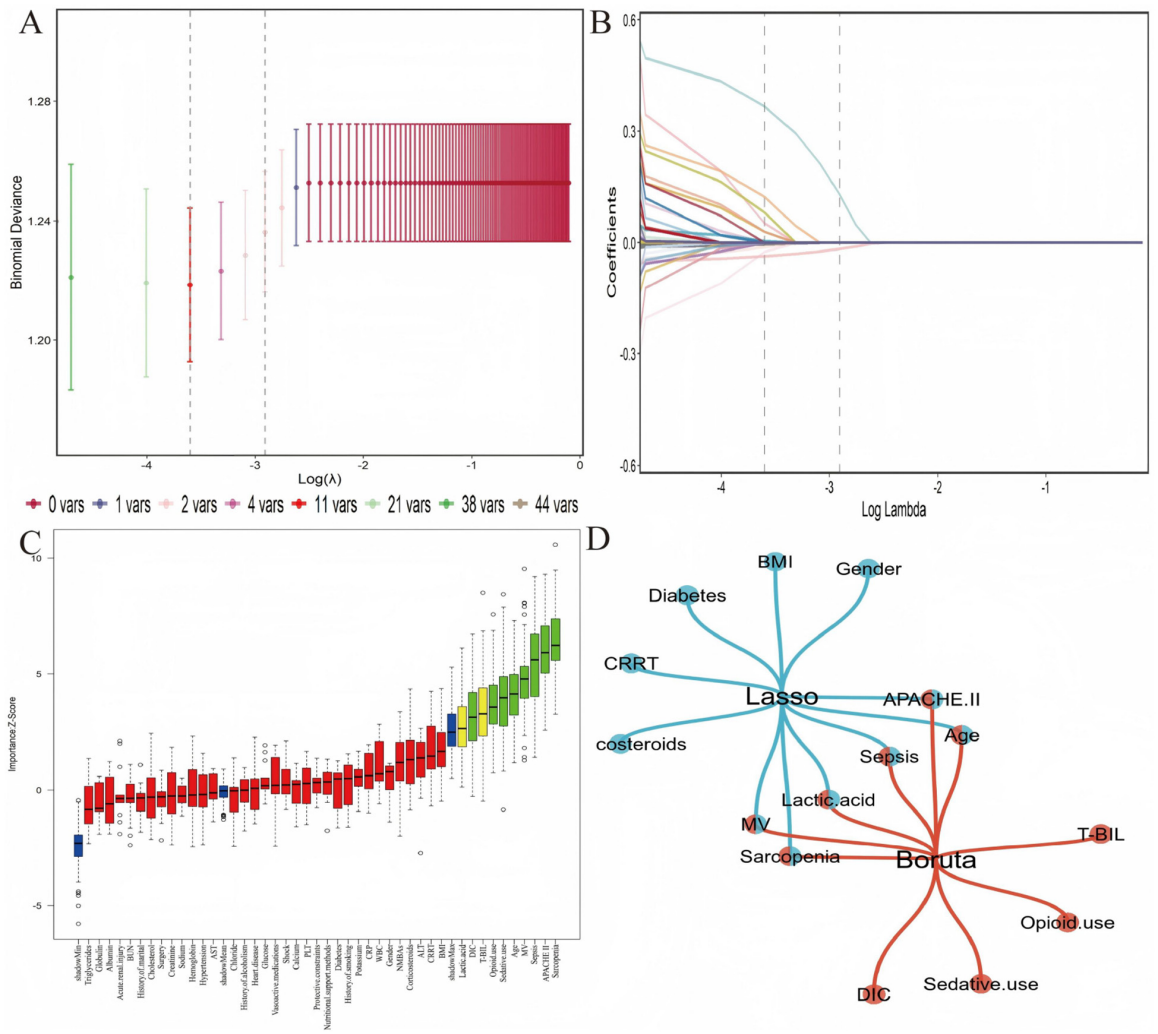
Feature selection using LASSO regression and Boruta. **(A)** LASSO coefficient profiles; **(B)** partial likelihood deviance plot with optimal lambda; **(C)** Boruta variable importance plot; **(D)** intersection of factors screened by LASSO regression and Boruta algorithm, identifying 6 core variables for ICU-AW.

Six influencing factors for ICU-AW were identified by taking the intersection of the results from LASSO regression and Boruta algorithm, which were Age, APACHE II score, sarcopenia, sepsis, MV and lactic acid (Figure 2). The correlation coefficients among all these screened variables were less than 0.4, indicating no significant correlation and the absence of multicollinearity among the variables (Supplementary Figure 2).

### Development and comparison of ten machine learning models in the training set

**Figure 3:**
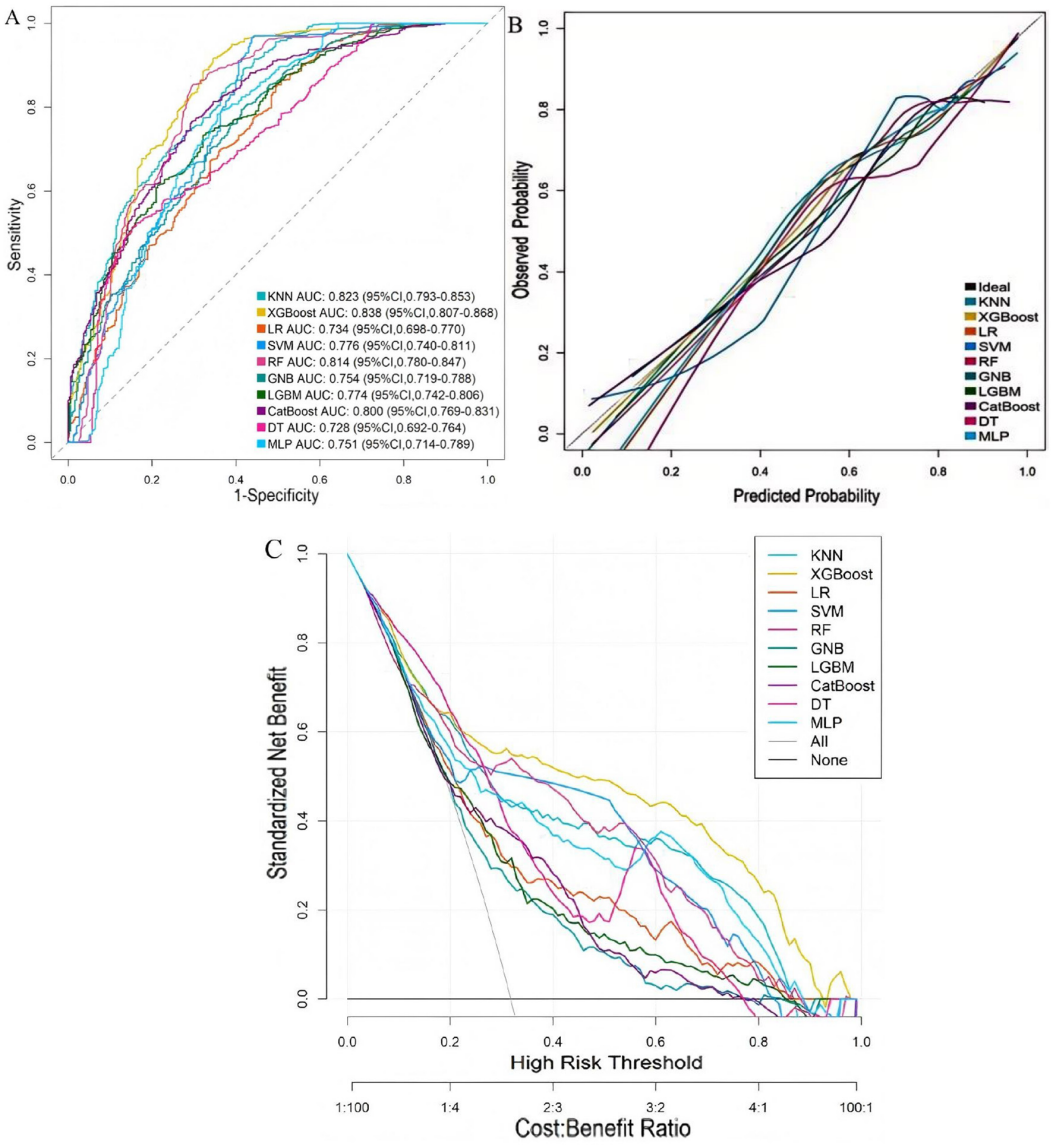
Performance comparison of 10 machine learning models in the training set. **(A)** ROC curves; **(B)** calibration curves; **(C)** decision curve analysis.

Based on the six selected predictors, 10 machine learning models were developed and compared in the training set. Among these, the XGBoost model exhibited the best overall predictive performance, achieving an AUC of 0.838 (95% CI: 0.807–0.868), a calibration slope of 0.901, and a calibration intercept of −0.030. It also demonstrated superior calibration, with the lowest Brier score of 0.137, and the decision curve analysis showed a net benefit across a threshold probability range of 8%−93%. The clinical impact curve further confirmed the model's stable predictive performance and its ability to effectively distinguish high-risk patients (Figure 3, Supplementary Figures 3, 4, Table 2).

**Table 2:** Performance metrics of 10 machine learning models in the training set.

Model	AUC	Sensitivity	Specificity	Accuracy	PPV	NPV	F1 score	Brier score	Calibration slope	Calibration intercept
KNN	0.823 (0.793–0.853)	0.891	0.583	0.793	0.821	0.714	0.855	0.182	0.842	−0.071
XGBoost	0.838 (0.807–0.868)	0.916	0.654	0.833	0.850	0.785	0.882	0.137	0.901	−0.030
LR	0.734 (0.698–0.770)	0.864	0.492	0.745	0.784	0.628	0.822	0.175	1.185	0.068
SVM	0.776 (0.740–0.811)	0.965	0.563	0.837	0.825	0.883	0.890	0.161	0.875	−0.047
RF	0.814 (0.780–0.847)	0.854	0.702	0.806	0.860	0.692	0.857	0.164	1.175	0.039
GNB	0.754 (0.719–0.788)	0.794	0.586	0.728	0.804	0.571	0.799	0.216	1.225	0.084
LGBM	0.774 (0.742–0.806)	0.733	0.678	0.715	0.830	0.542	0.778	0.188	0.834	−0.079
CatBoost	0.800 (0.769–0.831)	0.766	0.705	0.746	0.848	0.584	0.805	0.183	1.168	0.073
DT	0.728 (0.692–0.764)	0.514	0.847	0.620	0.878	0.449	0.648	0.251	1.378	0.118
MLP	0.751 (0.714–0.789)	0.783	0.644	0.739	0.825	0.581	0.803	0.239	1.342	0.108

KNN, k-nearest neighbor; XGBoost, extreme gradient boosting; LR, logistic regression; SVM, support vector machine; RF, random forest; GNB, gaussian naive bayes; LGBM, light gradient boosting machine; CatBoost, categorical boosting; DT, decision tree; MLP, multilayer perceptron.

### SHAP analysis for feature importance interpretation in the training set

**Figure 4:**
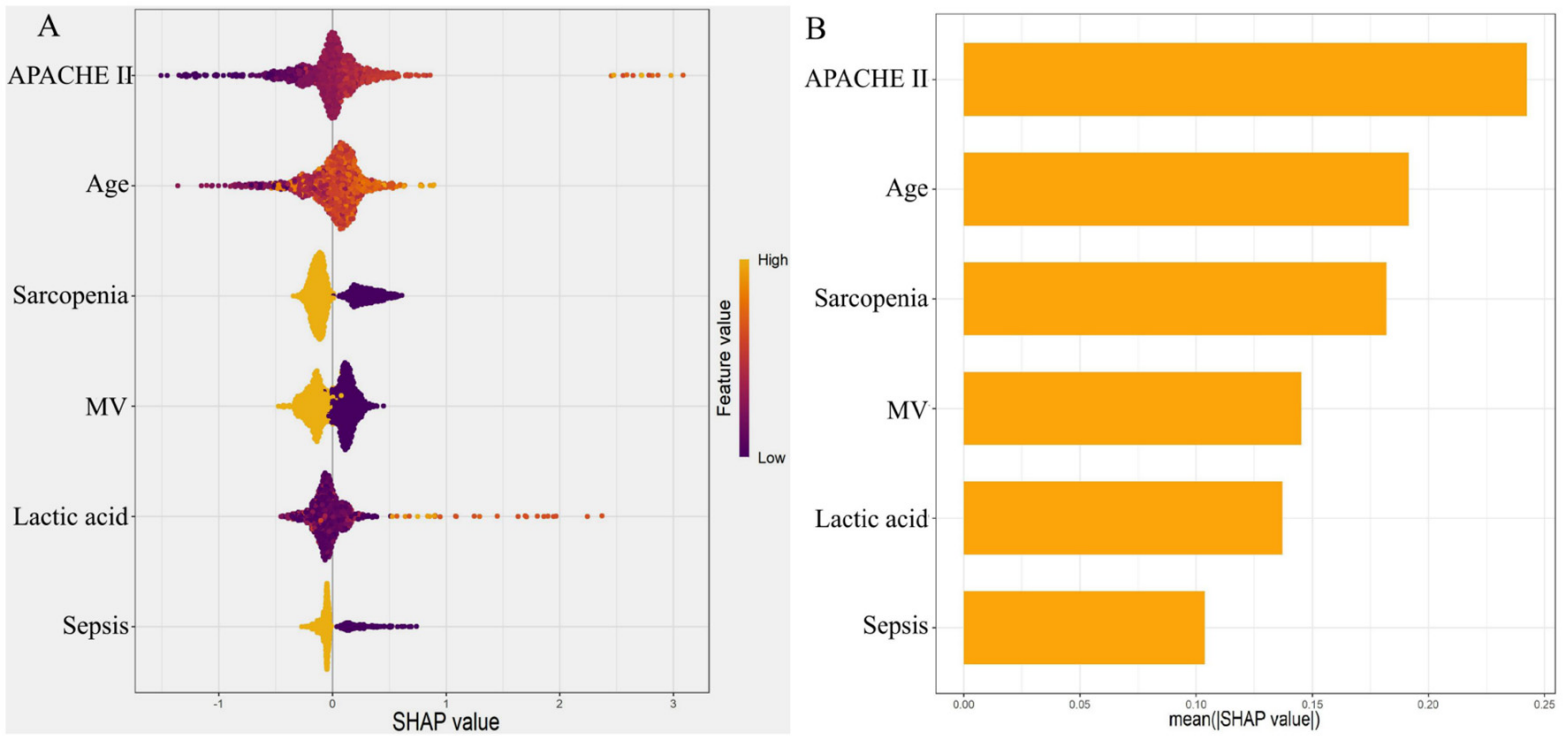
SHAP analysis illustrating the contribution of each feature to the model output. **(A)** SHAP summary plot (bee swarm plot); **(B)** SHAP feature importance bar plot.

To further interpret the XGBoost model, SHAP analysis was performed to quantify the contribution of each predictor. The ranked order of feature importance was as follows: APACHE II score, age, sarcopenia, MV, lactic acid, and sepsis (Figure 4 and Supplementary Figure 5).

### Validation of the XGBoost model in the validation set

**Figure 5:**
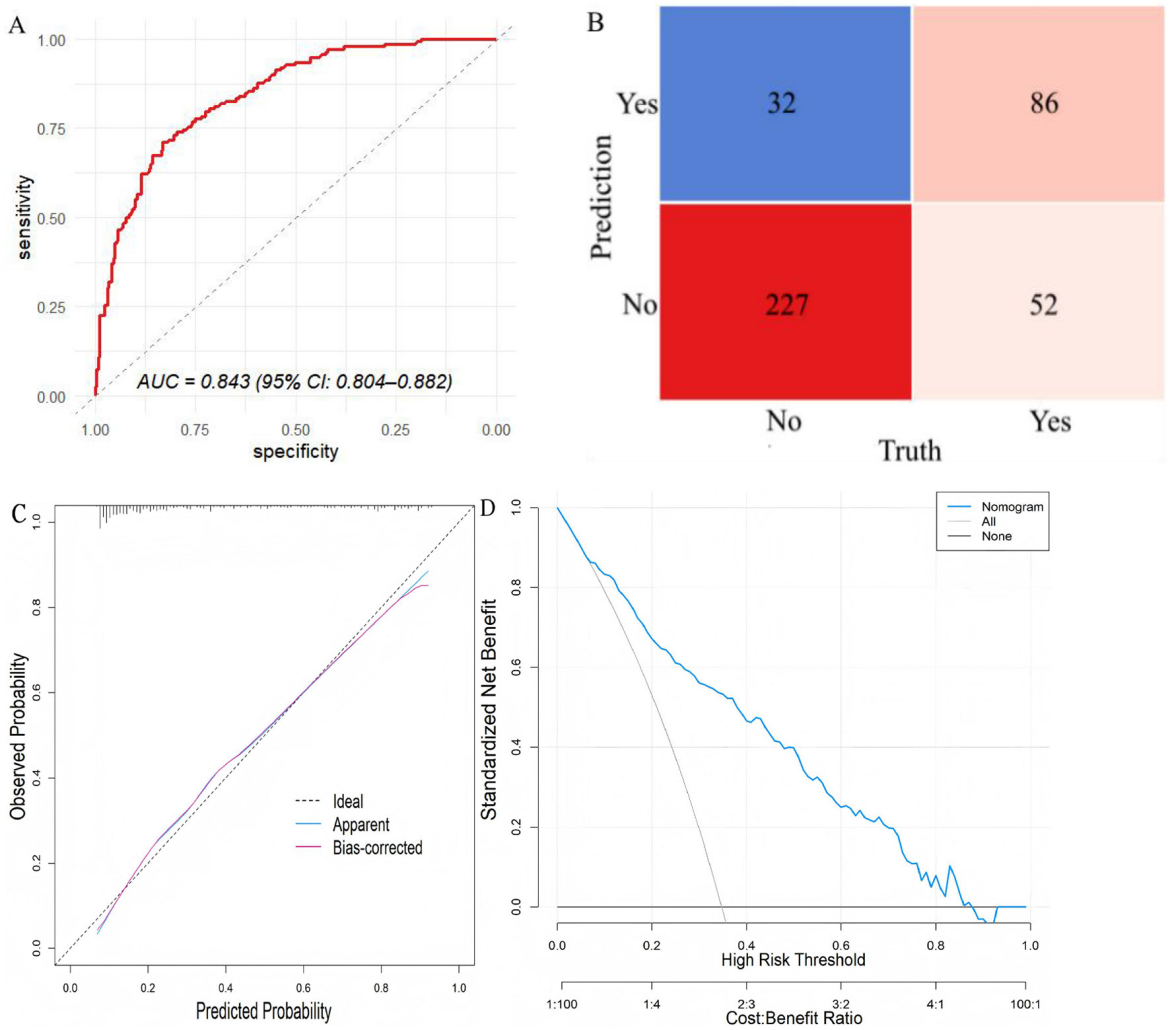
Performance of the XGBoost model in the validation set. **(A)** ROC curve; **(B)** confusion matrix; **(C)** calibration curve; **(D)** decision curve analysis.

The predictive performance of the XGBoost model was further evaluated in the validation set. The model achieved an accuracy of 0.788, with a sensitivity of 0.623 and a specificity of 0.876. The AUC was 0.843 (95% CI: 0.804–0.882), indicating good discriminative ability. As shown in Supplementary Figure 6, the predicted probabilities for patients with ICU-AW were substantially higher than those for patients without ICU-AW. The calibration curve demonstrated satisfactory agreement between predicted and observed outcomes, with a Brier score of 0.151, a calibration slope of 0.889, and a calibration intercept of 0.039. Decision curve analysis revealed a positive net benefit across a threshold probability range of 9%−92%, and the clinical impact curve further confirmed the model's clinical utility in identifying high-risk patients (Figure 5 and Supplementary Figure 7).

### Comparison with a simple LR model

To assess whether the added complexity of the XGBoost model provides clinically meaningful improvement over a transparent and easily deployable LR model, we compared their predictive performance in the validation set using the same six predictors. The LR model achieved an AUC of 0.752 (95% CI: 0.702–0.802), a Brier score of 0.231, a calibration slope of 0.804, and a calibration intercept of 0.098. Its ROC curve, calibration curve, and decision curve analysis are presented in Supplementary Figure 8. The difference in AUC between XGBoost (0.843) and LR (0.752) was statistically significant (*Z* = 6.2748, *P* < 0.001). Although the XGBoost model demonstrated superior discriminative ability, the LR model still provides acceptable performance and may be easier to implement in resource-limited settings. To facilitate clinical application, we developed an online dynamic nomogram based on the LR model, accessible at: https://fjzlzp.shinyapps.io/icuaw/ (Supplementary Figure 9).

## Discussion

In our cohort of 1,324 critically ill patients, the overall incidence of ICU-AW was 32.7%. Using LASSO regression and the Boruta algorithm, we identified six key predictors: age, APACHE II score, sarcopenia, sepsis, mechanical ventilation, and lactic acid. Among 10 machine learning models, the XGBoost model achieved the best predictive performance, with an AUC of 0.838 in the training set and 0.843 in the validation set. SHAP analysis revealed that sarcopenia ranked as the third most important predictor, following APACHE II score and age.

The pathogenesis of ICU-AW is driven by synergistic multifactorial mechanisms. Systemic inflammation, characterized by elevated levels of cytokines such as TNF-α and IL-6, activates the ubiquitin-proteasome system and suppresses mTOR signaling, thereby triggering rapid skeletal muscle catabolism (36). Concurrently, prolonged immobilization and metabolic stress induce mitochondrial dysfunction, leading to excessive oxidative damage and the activation of ferroptosis—an iron-dependent cell death pathway increasingly recognized as a key contributor to sepsis-associated myopathy (37, 38). Furthermore, emerging evidence highlights central nervous system involvement, including reduced corticospinal drive and neuroinflammation, positioning ICU-AW as a dual peripheral-central neuromuscular disorder (39). These interconnected mechanisms provide a framework for understanding the development and progression of ICU-AW in critically ill patients.

As a well-recognized component and biological precursor of physical frailty, sarcopenia predisposes patients to a pre-existing state of muscular vulnerability, characterized by reduced muscle mass, impaired contractile function, and disrupted protein metabolism (19, 40, 41). Sarcopenia is increasingly recognized as the biological substrate of physical frailty and a key component or precursor of the frailty syndrome (25). Both conditions exhibit substantial overlap in underlying mechanisms, including chronic inflammation, mitochondrial dysfunction, insulin resistance, dysregulation of protein homeostasis, and impaired stress adaptation (42, 43). In the context of critical illness, sarcopenia likely represents a state of diminished physiological reserve and heightened vulnerability to catabolic insults, thereby directly contributing to the development and severity of ICU-AW.

From a clinical perspective, SHAP analysis shows that sarcopenia, sepsis, mechanical ventilation, and lactic acid are potentially modifiable through targeted interventions (e.g., nutritional support, infection control, early mobilization, and hemodynamic optimization), whereas age and APACHE II score are non-modifiable but useful for early risk stratification. Based on our decision curve analysis, a risk threshold of approximately 20%−30% offers a favorable net benefit, and we suggest that patients exceeding this threshold receive enhanced preventive measures. The APACHE II score reflects the overall severity of critical illness, with higher scores indicating greater physiological derangement and systemic inflammatory burden, both of which accelerate muscle proteolysis and neuromuscular dysfunction (44). Advanced age is associated with diminished physiological reserve, age-related muscle loss, and impaired regenerative capacity, rendering older patients more susceptible to ICU-AW (32). MV contributes to diaphragmatic dysfunction and prolonged immobilization, promoting disuse atrophy and contractile dysfunction of both respiratory and limb muscles (30). Elevated lactic acid levels signify tissue hypoperfusion and bioenergetic failure, reflecting mitochondrial dysfunction and oxidative stress that impair muscle energy metabolism and exacerbate catabolism (31, 45). Sepsis, a well-established driver of ICU-AW, triggers a systemic inflammatory cascade characterized by excessive cytokine release, activation of the ubiquitin-proteasome pathway, and suppression of protein synthesis, leading to rapid and profound muscle wasting (46, 47).

Although previous studies have established several predictive models for ICU-AW, most have relied on traditional logistic regression or conventional machine learning algorithms, with only moderate predictive performance and without incorporating sarcopenia as a predictor (44, 46, 48–53). In contrast, our study is the first to integrate sarcopenia into a machine learning-based risk stratification tool. Our XGBoost model demonstrated superior discriminative ability compared to these prior models. Additionally, our model exhibited excellent calibration (Brier score 0.137) and stable clinical utility across a wide threshold probability range (9%−92%), as confirmed by decision curve analysis.

It is noteworthy that the sensitivity of the XGBoost model dropped from 0.916 in the training set to 0.623 in the validation set, meaning that about 37.7% of ICU-AW patients in the validation set were missed. This decline may be explained by residual class imbalance (despite class weight adjustment), possible overfitting, and differences in case mix between the two sets. Clinically, the reduced sensitivity indicates that although the model is highly specific (0.876), it may fail to identify many high-risk patients who could benefit from early intervention. The XGBoost model enables early identification of high-risk patients at ICU admission, assisting clinicians in implementing targeted preventive strategies, such as individualized nutritional support, early mobilization protocols, and close monitoring of muscle function. These recommendations are consistent with current clinical guidelines, including the American Thoracic Society guideline on the diagnosis of ICU-AW, both of which emphasize the importance of early rehabilitation and nutritional support for at-risk patients (15, 17). However, the XGBoost model may require specialized computational environments, which may limit its immediate bedside application in resource-limited settings. As an alternative, we developed an online dynamic nomogram based on the logistic regression model, which uses the same six predictors and is freely accessible via a web browser. Although the discriminative ability of the logistic regression model is slightly lower than that of XGBoost, its performance remains acceptable. This nomogram provides a user-friendly, no-installation tool that can be used at the bedside or in outpatient clinics, thereby facilitating risk stratification even when advanced machine learning platforms are unavailable. Therefore, the choice between the XGBoost model and the logistic regression-based nomogram involves a trade-off between maximum predictive accuracy and practical deployability.

Several limitations of this study should be acknowledged. First, as a single-center retrospective study conducted in a specialized oncology hospital, the generalizability of our findings is limited. The high prevalence of sarcopenia and ICU-AW in this cancer population likely differs from general ICUs, constraining model transferability to non-oncology settings. Lack of external validation further reduces model robustness. Second, sarcopenia assessment relied on abdominal CT obtained within 48 h of ICU admission; however, not all critically ill patients undergo abdominal CT. In our study, 289 patients were excluded specifically due to unavailability of CT imaging. This criterion may systematically select patients with more severe conditions or specific clinical indications for CT, introducing selection bias and limiting the model's applicability to ICUs where routine admission CT is not performed. Third, the SMI cutoffs used to define sarcopenia were derived from community-dwelling Asian populations, and currently there are no universally accepted cutoffs specifically for critically ill patients. Acute fluid shifts, edema, and altered body composition in the ICU setting may affect CT-derived muscle area measurements. Additionally, survivor bias and selection bias were introduced due to the mandatory patient cooperation required for the MRC sum score in diagnosing ICU-AW. Patients who died before the assessment window (ICU day 7) and those with sedation or delirium were unable to complete the muscle strength evaluation, which excludes the most critically ill patients. Consequently, our model predicts ICU-AW conditional on survival to day 7 rather than the unconditional risk in the entire ICU population, and the reported incidence of 32.7% reflects only patients who survived to assessment. Because death precludes the occurrence of ICU-AW as defined, death and ICU-AW are competing events—a structure that standard binary prediction does not account for, and a competing risks model (e.g., Fine-Gray) cannot be applied given that ICU-AW status is indeterminable in non-survivors.

## Conclusion

This study demonstrates that sarcopenia is an important predictor of ICU-acquired weakness in critically ill patients. By integrating sarcopenia into a machine learning-based risk stratification tool, the XGBoost model exhibited excellent predictive performance and clinical utility. This model enables early identification of high-risk patients at ICU admission, facilitating timely implementation of targeted preventive interventions.

## Data Availability

The original contributions presented in the study are included in the article/Supplementary material, further inquiries can be directed to the corresponding authors.

## References

[1] Renner C Jeitziner M-M Albert M Brinkmann S Diserens K Dzialowski I . Guideline on multimodal rehabilitation for patients with post-intensive care syndrome. Crit Care. (2023) 27:301. doi: 10.1186/s13054-023-04569-5 37525219 PMC10392009

[2] Hiser SL Fatima A Ali M Needham DM. Post-intensive care syndrome (PICS): recent updates. J Intensive Care. (2023) 11:23. doi: 10.1186/s40560-023-00670-7 37221567 PMC10202754

[3] Vanhorebeek I Latronico N Van den Berghe G. ICU-acquired weakness. Intensive Care Med. (2020) 46:637–53. doi: 10.1007/s00134-020-05944-4 32076765 PMC7224132

[4] Xiaojie Z Zixuan W Jing W Wu F Xia L Shi S . Risk factors, diagnostic challenges, and emerging therapeutic strategies for ICU-acquired weakness: a brief review. J Multidiscip Healthc. (2025) 18:7769–78. doi: 10.2147/JMDH.S560139 41340877 PMC12671084

[5] Sabrina E Parry Selina M Tessa B Lynch GS Bongetti AJ Ridley EJ . The intensive care medicine research agenda for the management of ICU acquired weakness: a multinational, interprofessional perspective. Intensive Care Med. (2025) 51:2199–212. doi: 10.1007/s00134-025-08186-4 41182389

[6] Thille AW Boissier F Muller M Levrat A Bourdin G Rosselli S . Role of ICU-acquired weakness on extubation outcome among patients at high risk of reintubation. Crit Care. (2020) 24:86. doi: 10.1186/s13054-020-2807-9 32164739 PMC7069045

[7] Meyer-Frießem CH Malewicz NM Rath S Ebel M Kaisler M Tegenthoff M . Incidence, time course and influence on quality of life of intensive care unit-acquired weakness symptoms in long-term intensive care survivors. J Intensive Care Med. (2021) 36:1313–22. doi: 10.1177/0885066620949178 32799703

[8] Saccheri C Morawiec E Delemazure J Mayaux J Dubé BP Similowski T . ICU-acquired weakness, diaphragm dysfunction and long-term outcomes of critically ill patients. Ann Intensive Care. (2020) 10:1. doi: 10.1186/s13613-019-0618-4 31900667 PMC6942110

[9] Zhou W Yu L Fan Y Shi B Wang X Chen T . Effect of early mobilization combined with early nutrition on acquired weakness in critically ill patients (EMAS): a dual-center, randomized controlled trial. PLoS ONE. (2022) 17:e026. doi: 10.1371/journal.pone.0268599 35617287 PMC9135241

[10] Nakanishi N Yoshihiro S Kawamura Y Aikawa G Shida H Shimizu M . Effect of neuromuscular electrical stimulation in patients with critical illness: an updated systematic review and meta-analysis of randomized controlled trials. Crit Care Med. (2023) 51:1386–96. doi: 10.1097/CCM.0000000000005941 37232695

[11] Pierre S Reintam BA Berger Mette M Calder Philip C Michael C Michael H . ESPEN practical and partially revised guideline: clinical nutrition in the intensive care unit. Clin Nutr. (2023) 42:1671–89. doi: 10.1016/j.clnu.2023.07.011 37517372

[12] Zhang H Sheng Y Li Q Li Z Luo H Zhang Q . Patient-centred approaches to prevent ICU-acquired weakness: insights from early mobilisation participation. Nurs Crit Care. (2025) 30:e70158. doi: 10.1111/nicc.70158 40878886

[13] Sunaryo EYAB Lee H Sofia L Peng Z Tsai H Lee W. Preventing ICU-acquired weakness with early rehabilitation: an umbrella review of systematic reviews and meta-analysis. Nurs Crit Care. (2025) 30:e70113. doi: 10.1111/nicc.70113 40717664

[14] Formenti P Menozzi A Sabbatini G Gotti M Galimberti A Bruno G . Combined effects of early mobilization and nutrition on ICU-acquired weakness. Nutrients. (2025) 17:1073. doi: 10.3390/nu17061073 40292494 PMC11945635

[15] Fan E Cheek F Chlan L Gosselink R Hart N Herridge MS . An official american thoracic society clinical practice guideline: the diagnosis of intensive care unit-acquired weakness in adults. Am J Respir Crit Care Med. (2014) 190:1437–46. doi: 10.1164/rccm.201411-2011ST 25496103

[16] Xi B Chunmei G Yanli L Xiaobo J Jinmei L Chuan G. Best evidence summary on early exercise for prevention of ICU-acquired weakness: an evidence-based synthesis. J Multidiscip Healthc. (2026) 19:57. doi: 10.2147/JMDH.S574831 41737368 PMC12927820

[17] Girard TD Alhazzani W Kress JP Ouellette DR Schmidt GA Truwit JD . An official American thoracic society/American college of chest physicians clinical practice guideline: liberation from mechanical ventilation in critically ill adults. rehabilitation protocols, ventilator liberation protocols, and cuff leak tests. Am J Respir Crit Care Med. (2017) 195:120–33. doi: 10.1164/rccm.201610-2075ST 27762595

[18] Yu LR Jia WJ Tian WM Cha HT Yong JJ. Optimal timing for early mobilization initiatives in intensive care unit patients: a systematic review and network meta-analysis. Intensive Crit Care Nurs. (2024) 82:10(3607) doi: 10.1016/j.iccn.2023.103607 38158250

[19] Sayer AA Cruz-Jentoft A. Sarcopenia definition, diagnosis and treatment: consensus is growing. Age Ageing. (2022) 51:afac220. doi: 10.1093/ageing/afac220 36273495 PMC9588427

[20] Qu G Zhou C Zhang Y Lyu S Lang R. Influence of sarcopenia on postoperative complications and long-term survival in pancreatic cancer patients undergone pancreaticoduodenectomy. Front Nutr. (2024) 11:1434630. doi: 10.3389/fnut.2024.1434630 39027658 PMC11254807

[21] Qu J Liu Y Yuan Y Yu Z Ding J He Z . Impacts of sarcopenia on adverse events and prognosis in Chinese patients with esophageal cancer undergoing chemoradiotherapy. Front Nutr. (2025) 12:1523674. doi: 10.3389/fnut.2025.1523674 40051963 PMC11882421

[22] Luo J Yang D Xu Z Zhang D Li M Kong Y . A prospective study on the differential association of sarcopenia and frailty with health outcomes in cirrhotic patients. Dig Liver Dis. (2023) 55:1533–42. doi: 10.1016/j.dld.2023.07.007 37482521

[23] Wang C Guo X Xu X Liang S Wang W Zhu F . Association between sarcopenia and frailty in elderly patients with chronic kidney disease. J Cachexia Sarcopenia Muscle. (2023) 14:1855–64. doi: 10.1002/jcsm.13275 37300354 PMC10401549

[24] Kosoku A Iwai T Kabei K Nishide S Machida Y Uchida J. Frailty and sarcopenia in older kidney transplant recipients: a cross-sectional study. Eur Geriatr Med. (2023) 14:861–8. doi: 10.1007/s41999-023-00803-z 37219724

[25] Xinyu M Xia W Qingyue Z Xijie Y Shuangqing L. Sarcopenia and the frailty progression among Chinese: a longitudinal study. Front Public Health. (2025) 13:1551282. doi: 10.3389/fpubh.2025.1551282 41561850 PMC12812598

[26] Ma G Ma G Yu L Huang S Gao H. Analysis for risk factors for frailty syndrome in elderly patients with acute coronary syndrome and establishment of a nomogram prediction model. Cardiology. (2025) 15:1–11. doi: 10.1159/000548077 40947836

[27] Tieland M van Dronkelaar C Boirie Y. Sarcopenic obesity in the ICU. Curr Opin Clin Nutr Metab Care. (2019) 22:162–6. doi: 10.1097/MCO.0000000000000547 30585801

[28] van der Steen-Dieperink MJMM Koekkoek WAC Kouw IWK. Sarcopenia and frailty in critical illness. Curr Opin Clin Nutr Metab Care. (2025) 28:192–9. doi: 10.1097/MCO.0000000000001123 40072495 PMC11970596

[29] Li K Alhaskawi A Zhou H Dong Y Zhao Q Wang C . Risk factors and electromyographic characteristics of acquired weakness in critically ill patients: a retrospective study. Ther Clin Risk Manag. (2024) 20:451–63. doi: 10.2147/TCRM.S464722 39104821 PMC11299719

[30] Liu J Xu Z Luo S Bai Y Feng J Li F. Risk factors for ICU-acquired weakness in sepsis patients: a retrospective study of 264 patients. Heliyon. (2024) 10:e3. doi: 10.1016/j.heliyon.2024.e32253 PMC11168428 38867955

[31] Yang T Li Z Jiang L Xi X. Hyperlactacidemia as a risk factor for intensive care unit-acquired weakness in critically ill adult patients. Muscle Nerve. (2021) 64:77–82. doi: 10.1002/mus.27248 33831220

[32] Zheng Q Liu C Le L Wu Q Xu Z Lin J . ICU-acquired weakness in critically ill patients at risk of malnutrition: risk factors, biomarkers, and early enteral nutrition impact. World J Emerg Med. (2025) 16:51–6. doi: 10.5847/wjem.j.1920-8642.2025.020 39906116 PMC11788102

[33] Chen LK Woo J Assantachai P Auyeung TW Chou MY Iijima K . Asian working group for sarcopenia: 2019 consensus update on sarcopenia diagnosis and treatment. J Am Med Dir Assoc. (2020) 21:300–7. doi: 10.1016/j.jamda.2019.12.012 32033882

[34] Chen J Huang M. Intensive care unit-acquired weakness: Recent insights. J Intensive Med. (2024) 4:73–80. doi: 10.1016/j.jointm.2023.07.002 38263973 PMC10800771

[35] Guo Y Shan W Xiang J. Predictive modeling of ICU-AW inflammatory factors based on machine learning. BMC Neurol. (2024) 24:483. doi: 10.1186/s12883-024-03981-w 39702112 PMC11658462

[36] Marcela K Pavel K. Molecular mechanisms underlying intensive care unit-acquired weakness and sarcopenia. Int J Mol Sci. (2022) 23:8396. doi: 10.3390/ijms23158396 35955530 PMC9368893

[37] Klawitter F Ehler J Bajorat R Patejdl R. Mitochondrial dysfunction in intensive care unit-acquired weakness and critical illness myopathy: a narrative review. Int J Mol Sci. (2023) 24:5516. doi: 10.3390/ijms24065516 36982590 PMC10052131

[38] Yang J Yan C Chen S Min Li. Yanmei Miao, Xinglong Ma, et al. The possible mechanisms of ferroptosis in sepsis-associated acquired weakness. Front Physiol. (2024) 15:1380992. doi: 10.3389/fphys.2024.1380992 38601213 PMC11004370

[39] Souron R Morel J Gergelé L Infantino P Brownstein CG Lapole T . Relationship between intensive care unit-acquired weakness, fatigability and fatigue: what role for the central nervous system. J Crit Care. (2021) 62:101–10. doi: 10.1016/j.jcrc.2020.11.019 33316555

[40] Yuan S Larsson SC. Epidemiology of sarcopenia: Prevalence, risk factors, and consequences. Metabolism. (2023) 144:155533. doi: 10.1016/j.metabol.2023.155533 36907247

[41] Cassandra S Woessner Mary N Marc S Itamar L. Sarcopenia definition: does it really matter? implications for resistance training. Ageing Res Rev. (2022) 78:101617. doi: 10.1016/j.arr.2022.101617 35378297

[42] Hu Y Peng W Ren R Wang Y Wang G. Sarcopenia and mild cognitive impairment among elderly adults: the first longitudinal evidence from CHARLS. J Cachexia Sarcopenia Muscle. (2022) 13:2944–52. doi: 10.1002/jcsm.13081 36058563 PMC9745544

[43] Muñoz-Redondo E Morgado-Pérez A Pérez-Sáez MJ Pascual J Tejero-Sánchez M Curbelo YG . New perspectives on frailty in light of the global leadership initiative on malnutrition, the global leadership initiative on sarcopenia, and the WHO's concept of intrinsic capacity: a narrative review. Maturitas. (2023) 177:107799. doi: 10.1016/j.maturitas.2023.107799 37499428

[44] Lu C Wenjuan J. Construction and evaluation of acquired weakness nomogram model in patients with mechanical ventilation in intensive care unit. Digit Health. (2024) 10:2055207624126. doi: 10.1177/20552076241261604 PMC11271112 39055781

[45] Witteveen E Wieske L Sommers J Spijkstra J de Waard MC Endeman H . Early prediction of intensive care unit-acquired weakness: a multicenter external validation study. J Intensive Care Med. (2020) 35:595–605. doi: 10.1177/0885066618771001 29716425 PMC7222288

[46] Nakano FK Van Aerde N Coppens G Vens C Van den Berghe G Grandas FG. Development and validation of a machine learning model for early prediction of intensive care unit acquired weakness. Intensive Care Med Exp. (2025) 13:98. doi: 10.1186/s40635-025-00810-3 41026286 PMC12484466

[47] Jin C Hu F Wu L Zhang L Wu B Yang Y. Risk factors of ICU-acquired weakness in sepsis patients. Pak J Med Sci. (2025) 41:2587–94. doi: 10.12669/pjms.41.9.12558 41070322 PMC12505916

[48] Lei L He L Zou T Qiu J Li Y Zhou R . Predicting early diagnosis of intensive care unit-acquired weakness in septic patients using critical ultrasound and biological markers. BMC Anesthesiol. (2025) 25:39. doi: 10.1186/s12871-025-02911-8 39863865 PMC11761801

[49] Panda CK Karim HMR. Deep machine learning might aid in combating intensive care unit-acquired weakness. Cureus. (2024) 16:e58963. doi: 10.7759/cureus.58963 38800279 PMC11126887

[50] Yimei Z Yu W Jingran Y Li Q Zhou M Lu J . Development and validation of machine learning-based risk prediction models for ICU-acquired weakness: a prospective cohort study. Eur J Med Res. (2025) 30:666. doi: 10.1186/s40001-025-03356-y 40707986 PMC12288251

[51] Raurell-Torredà M Muriel-García A Arias-Rivera S. Predicting intensive care unit-acquired weakness in the first week of an intensive care unit stay: a multicentre external validation study. Aust Crit Care. (2025) 38:10. doi: 10.1016/j.aucc.2025.101241 40311514

[52] Yang Z Wang X Chang G Cao Q Wang F Peng Z . Development and validation of an intensive care unit acquired weakness prediction model: a cohort study. Front Med. (2023) 10:112. doi: 10.3389/fmed.2023.1122936 36910489 PMC9993479

[53] Wieske L Witteveen E Verhamme C Dettling-Ihnenfeldt DS van der Schaaf M Schultz MJ . Early prediction of intensive care unit-acquired weakness using easily available parameters: a prospective observational study. PLoS ONE. (2014) 9:e11. doi: 10.1371/journal.pone.0111259 PMC4210178 25347675

