# Supplementary material for: Integrating sarcopenia into ICU-acquired weakness risk stratification: a machine learning–based prediction model for critical care
Source: Front Nutr. 2026 May 14;13:1823112. doi: 10.3389/fnut.2026.1823112 (PMC13215805; doi:10.3389/fnut.2026.1823112)
Supplement: Supplementary file 1 [file Data_Sheet_1.pdf]

SUPPLEMENTARY TABLE 1 Optimal hyperparameters of the ten machine learning models.

Model	Hyperparameters
KNN	k = 5, weights = 'uniform', algorithm = 'auto'
XGBoost	n_estimators = 150, max_depth = 4, learning_rate = 0.05, subsample = 0.8, colsample_bytree = 0.8
LR	penalty = 'l2', C = 1.0, solver = 'lbfgs', max_iter = 1000
SVM	kernel = 'rbf', C = 1.0, gamma = 'scale', probability = True
RF	n_estimators = 200, max_depth = 5, min_samples_split = 5, min_samples_leaf = 2
GNB	var_smoothing = 1e-9 (default)
LGBM	n_estimators = 100, max_depth = 4, learning_rate = 0.05, subsample = 0.8, colsample_bytree = 0.8
CatBoost	iterations = 100, depth = 4, learning_rate = 0.05, l2_leaf_reg = 3
DT	max_depth = 5, min_samples_split = 5, min_samples_leaf = 2, criterion = 'gini'
MLP	hidden_layer_sizes = (50, 25), activation = 'relu', solver = 'adam', alpha = 0.0001, max_iter = 500

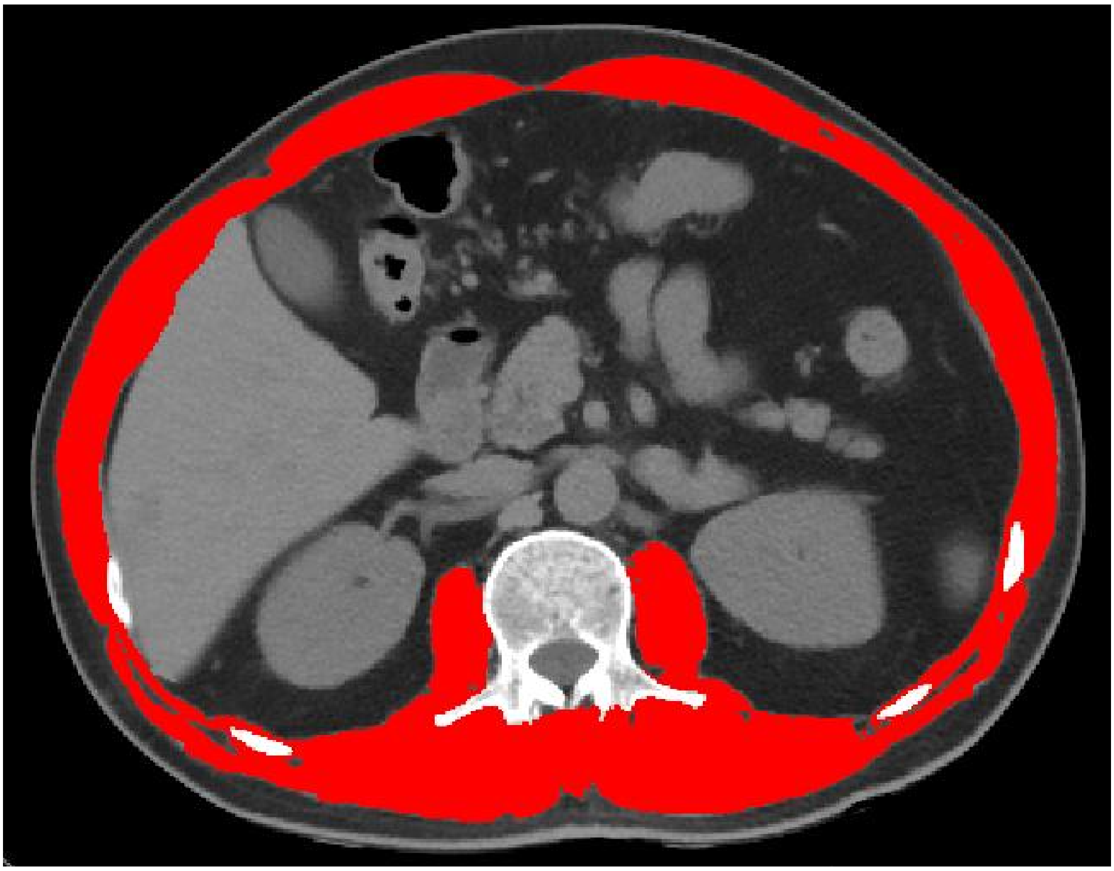

Supplementary Figure 1
Quantitative analysis of skeletal muscle area at the L3 level using CT imaging.

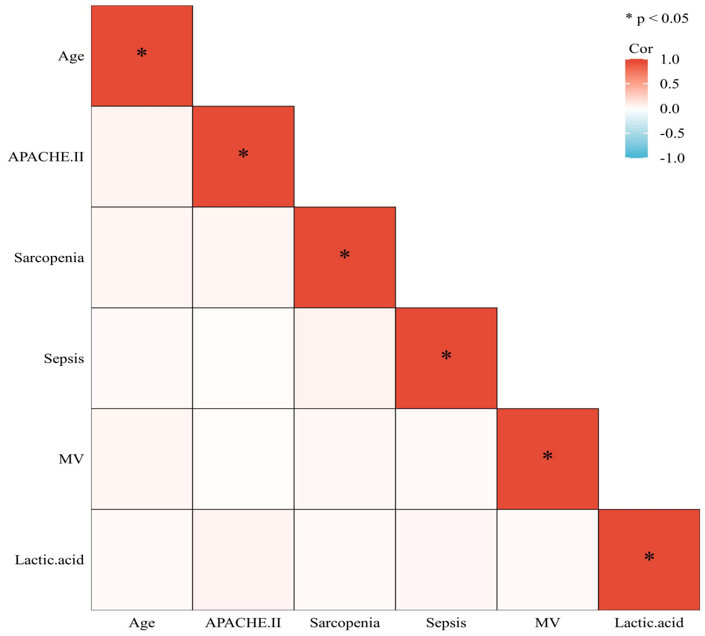

Supplementary Figure 2
Correlation heatmap of the six selected predictors.

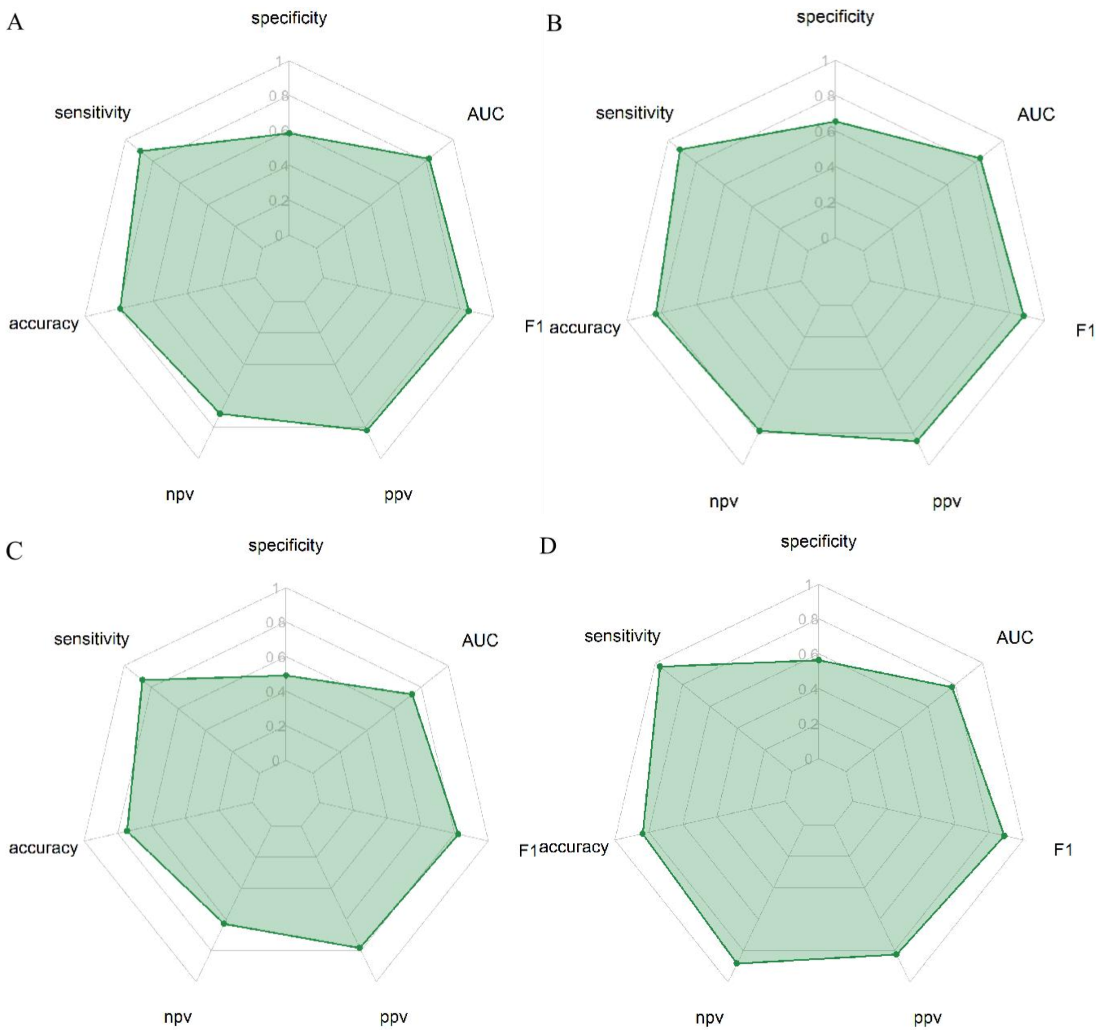

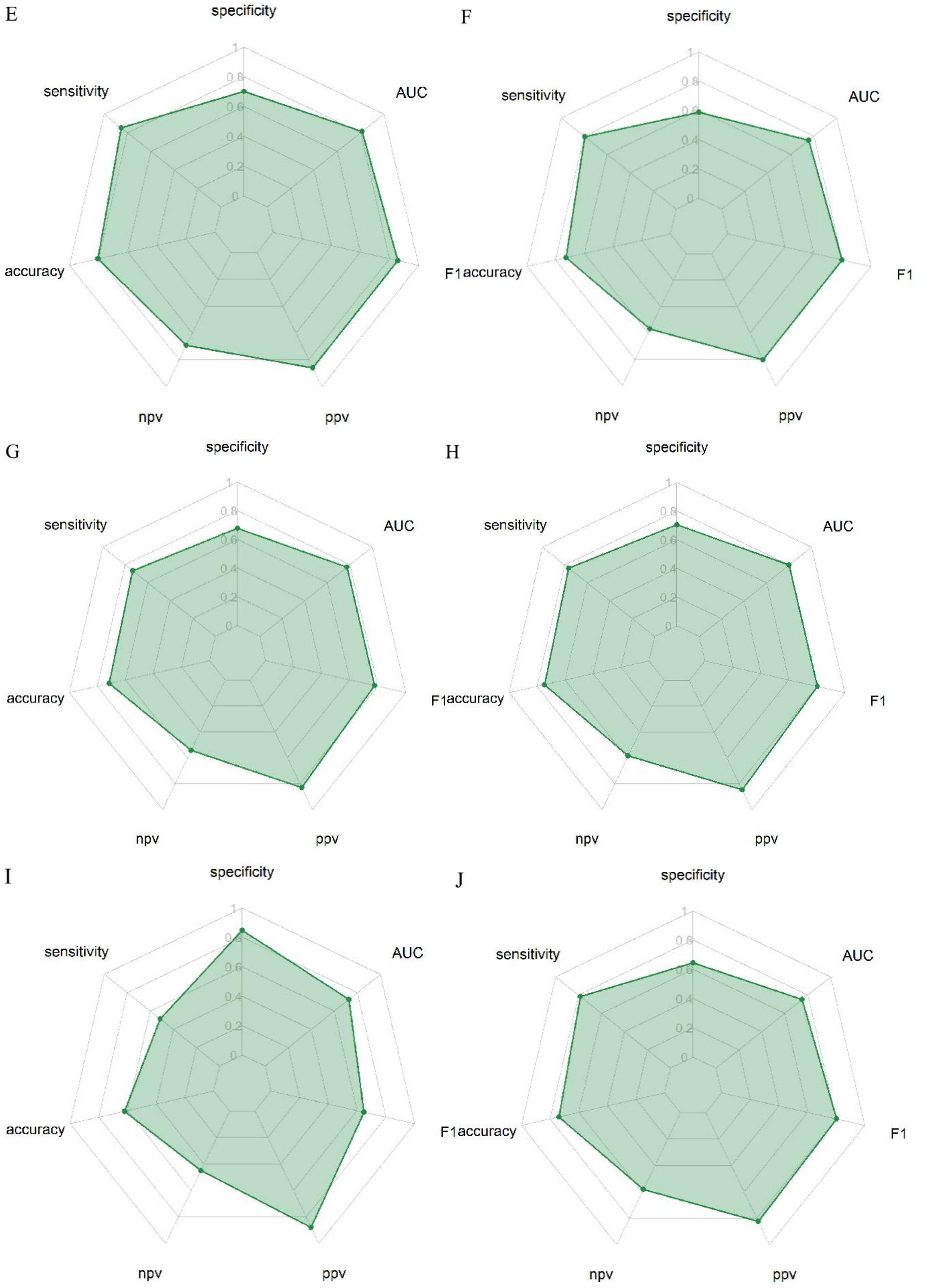

Supplementary Figure 3

Radar plots of ten machine learning models. (**A**) KNN; (**B**) XGBoost; (**C**) LR; (**D**) SVM; (**E**) RF; (**F**) GNB; (**G**) LGBM; (**H**) CatBoost; (**I**) DT; (**J**) MLP.

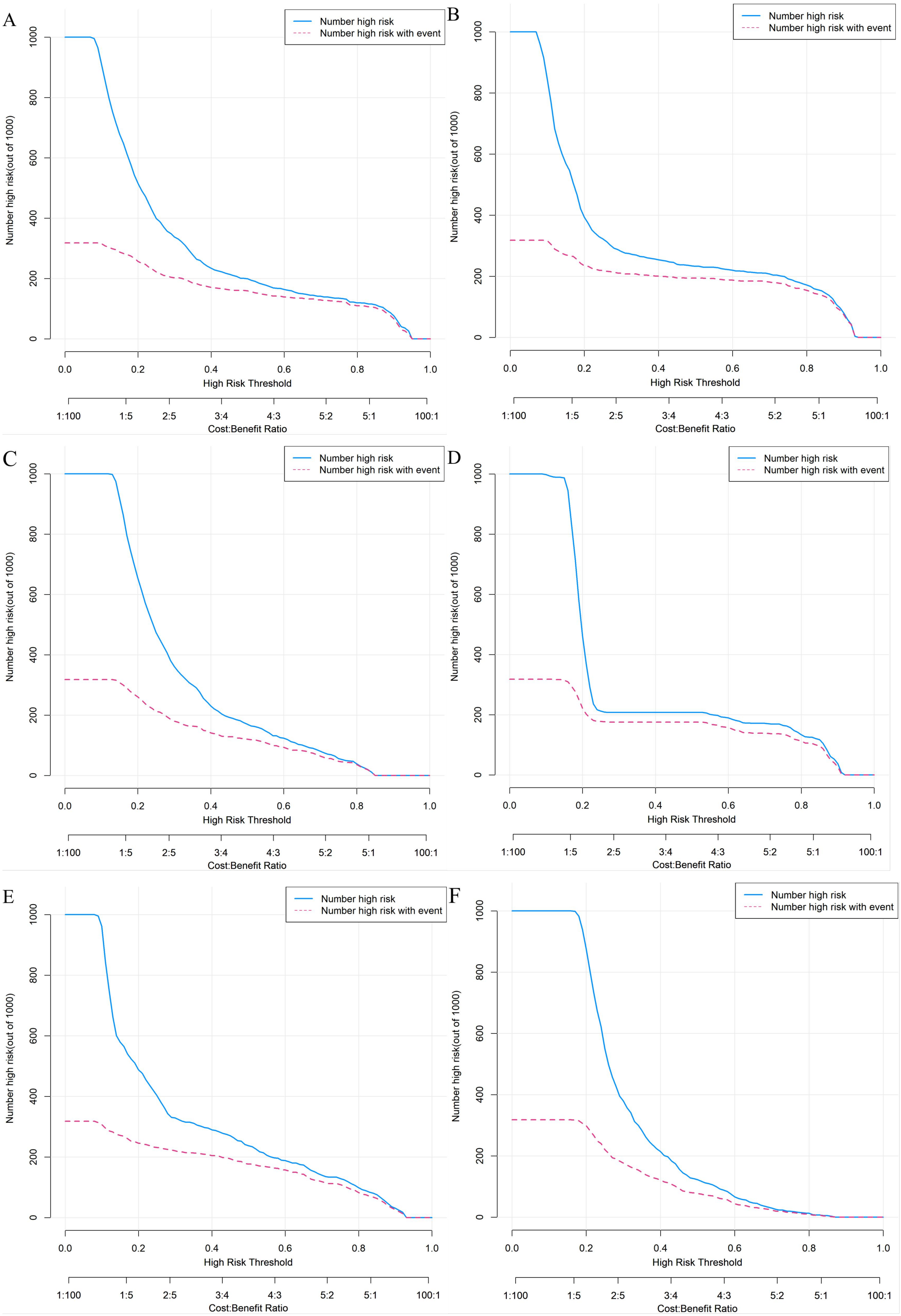
A
Number high risk
Number high risk with event
Number high risk(out of 1000)
0
200
400
600
800
1000
0.0
0.2
0.4
0.6
0.8
1.0
High Risk Threshold
1:100
1:5
2:5
3:4
4:3
5:2
5:1
100:1
Cost:Benefit Ratio
B
Number high risk
Number high risk with event
Number high risk(out of 1000)
High Risk Threshold
Cost:Benefit Ratio
C
Number high risk
Number high risk with event
Number high risk(out of 1000)
High Risk Threshold
Cost:Benefit Ratio
D
Number high risk
Number high risk with event
Number high risk(out of 1000)
High Risk Threshold
Cost:Benefit Ratio
E
Number high risk
Number high risk with event
Number high risk(out of 1000)
High Risk Threshold
Cost:Benefit Ratio
F
Number high risk
Number high risk with event
Number high risk(out of 1000)
High Risk Threshold
Cost:Benefit Ratio

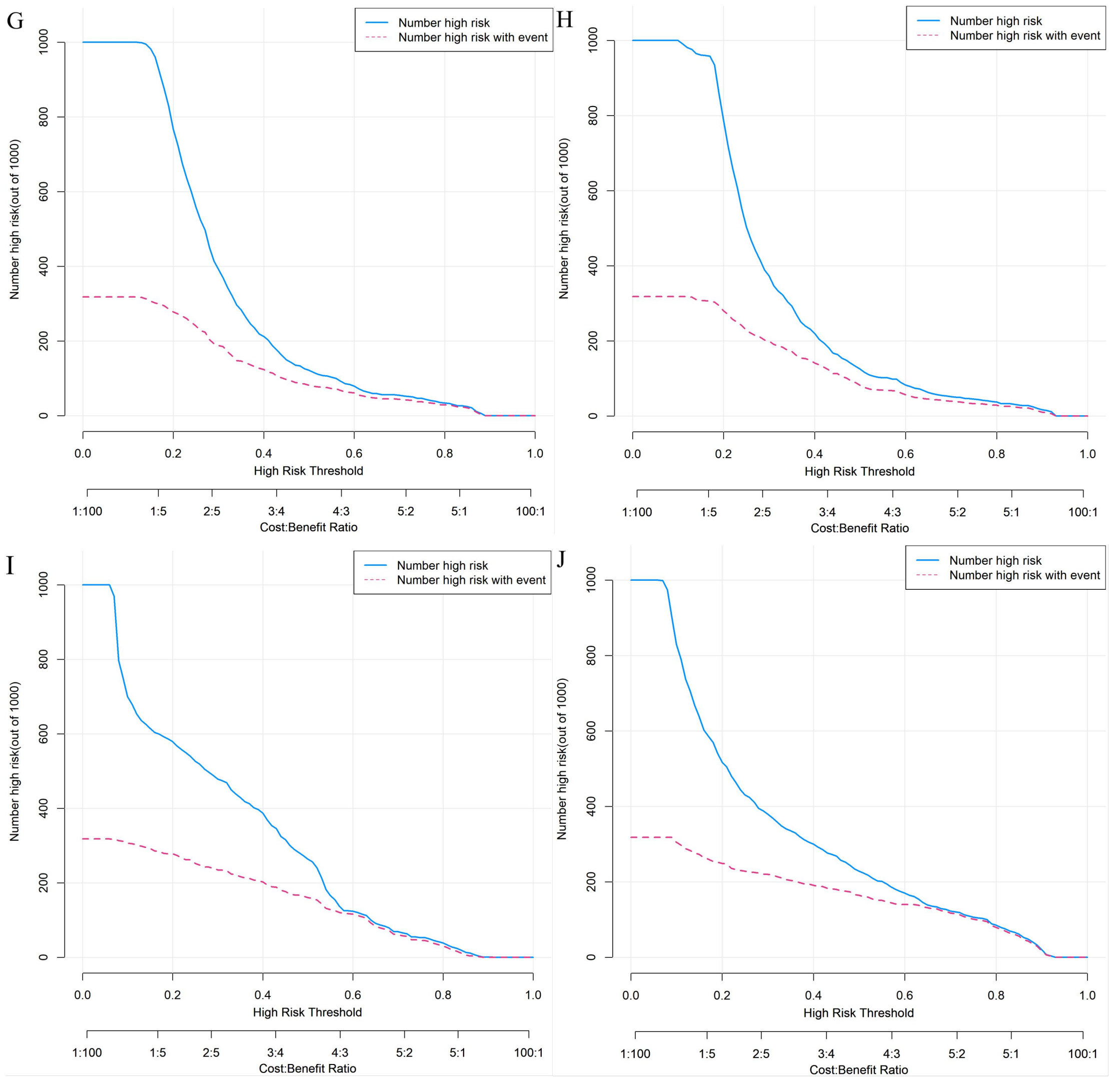

Supplementary Figure 4

Clinical impact curves of ten machine learning models. (**A**) KNN; (**B**) XGBoost; (**C**) LR; (**D**) SVM; (**E**) RF; (**F**) GNB; (**G**) LGBM; (**H**) CatBoost; (**I**) DT; (**J**) MLP.

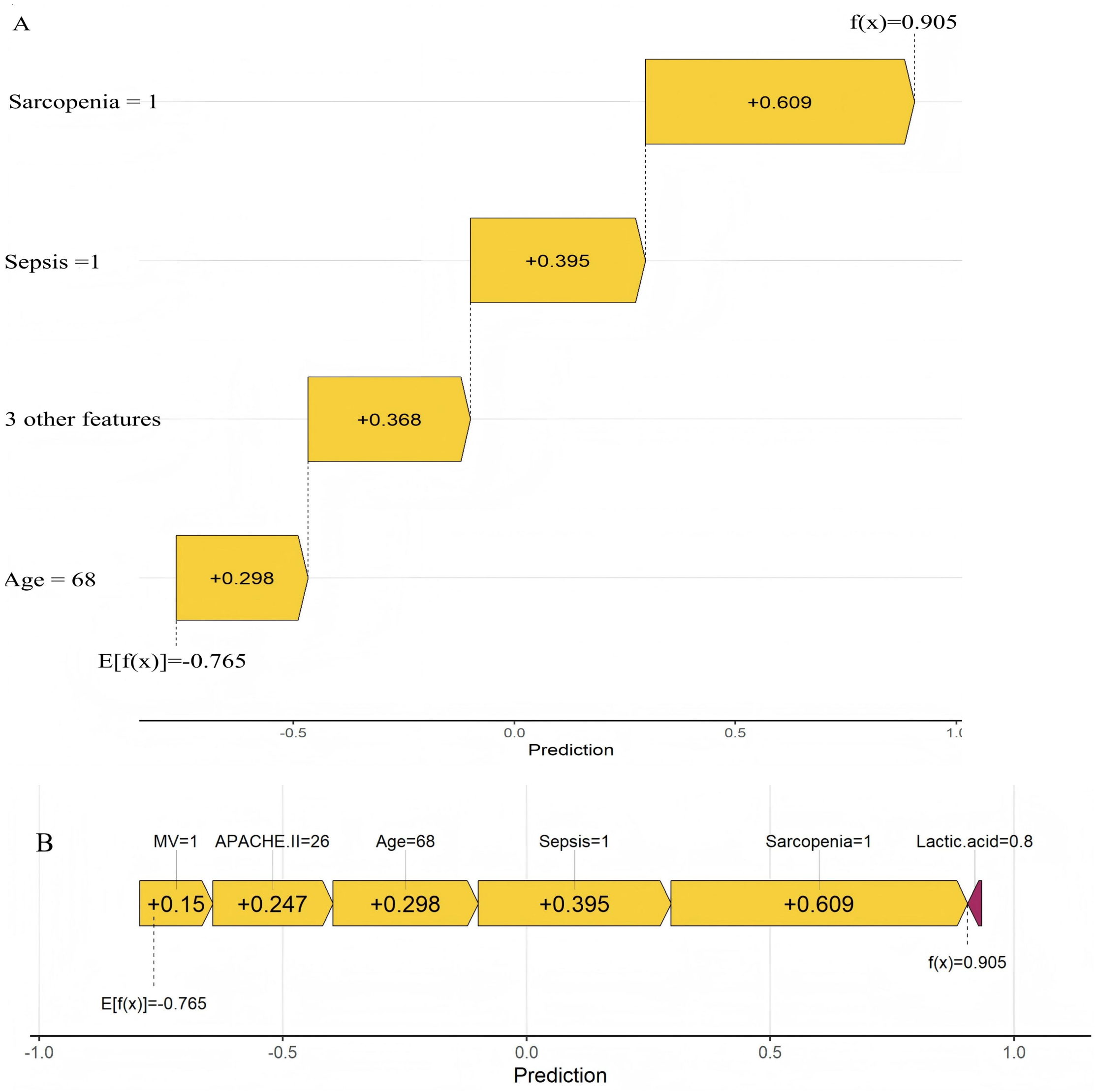

Supplementary Figure 5

Combined SHAP explanation for an individual prediction. (**A**) Waterfall plot and (**B**) force plot jointly illustrate how the prediction is formed for a representative high-risk patient (age = 68 years, APACHE II = 26, with sarcopenia and sepsis). The elevated risk is primarily driven by sarcopenia and sepsis, with both plots visualizing the direction and magnitude of each feature's contribution.

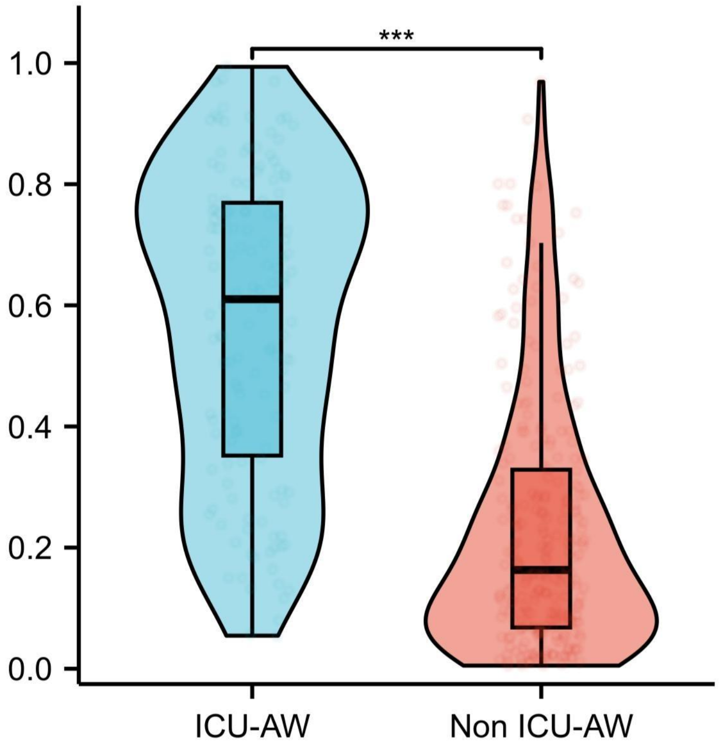

Supplementary Figure 6

Distribution of predicted probabilities between patients with and without ICU-AW.

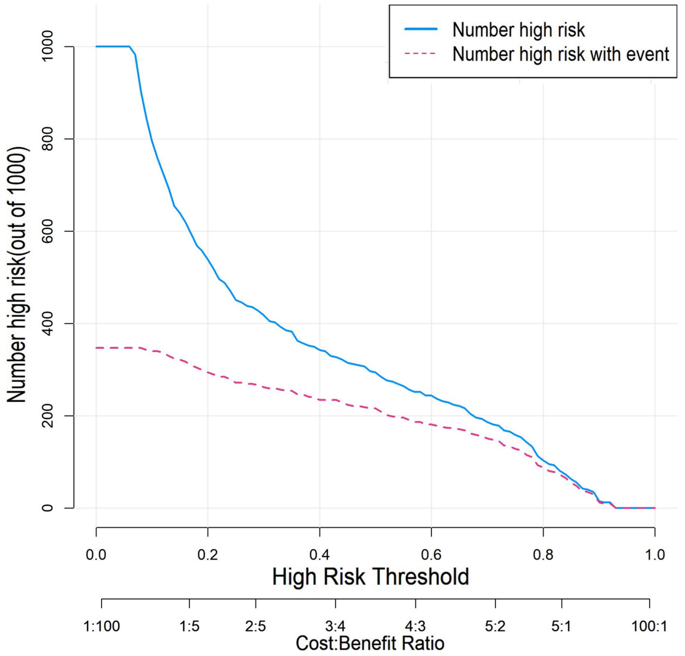

Supplementary Figure 7

Clinical impact curves of validation set.

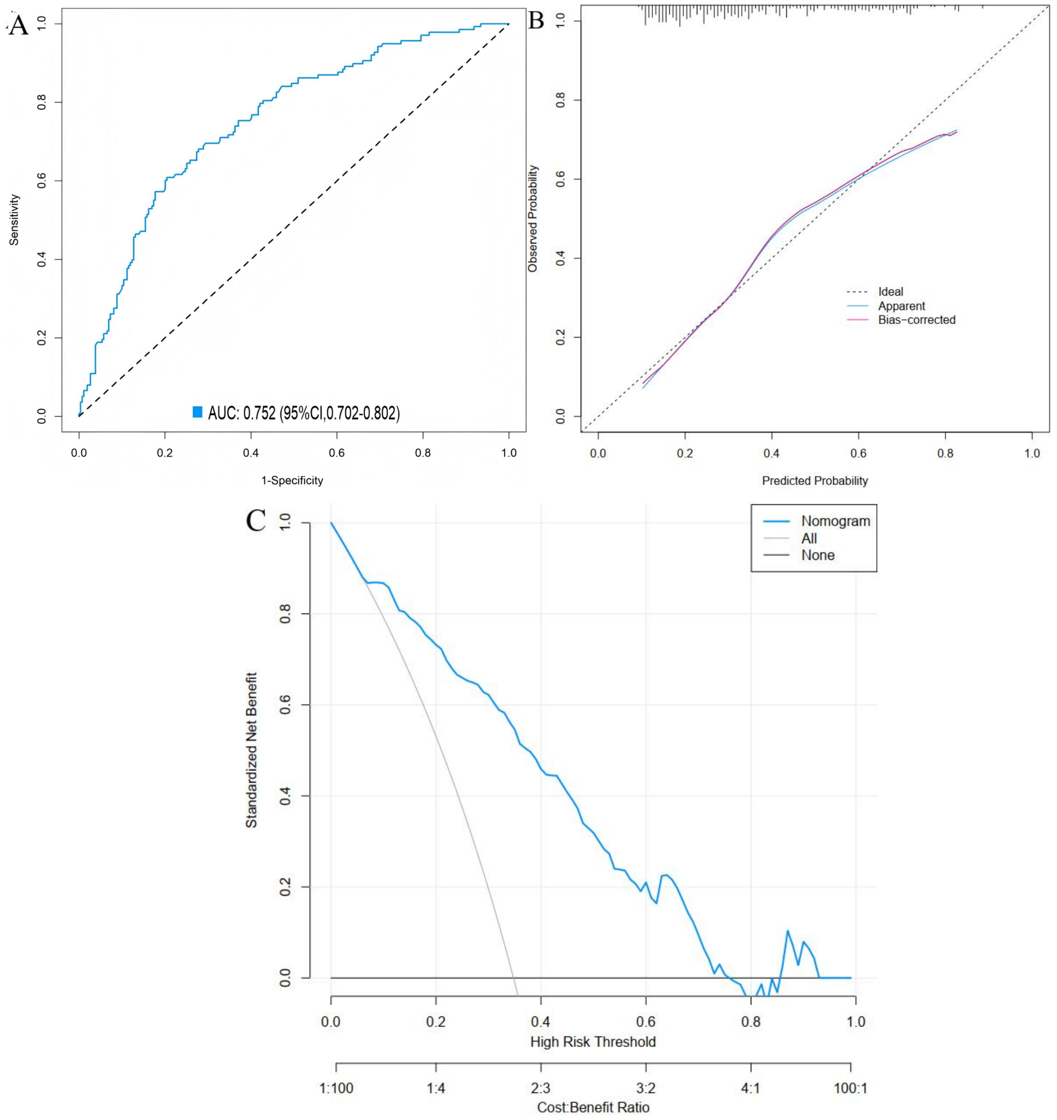

Supplementary Figure 8

Performance of the logistic regression model in the validation set. (**A**) ROC curve; (**B**) Calibration curve; (C) Decision curve analysis.

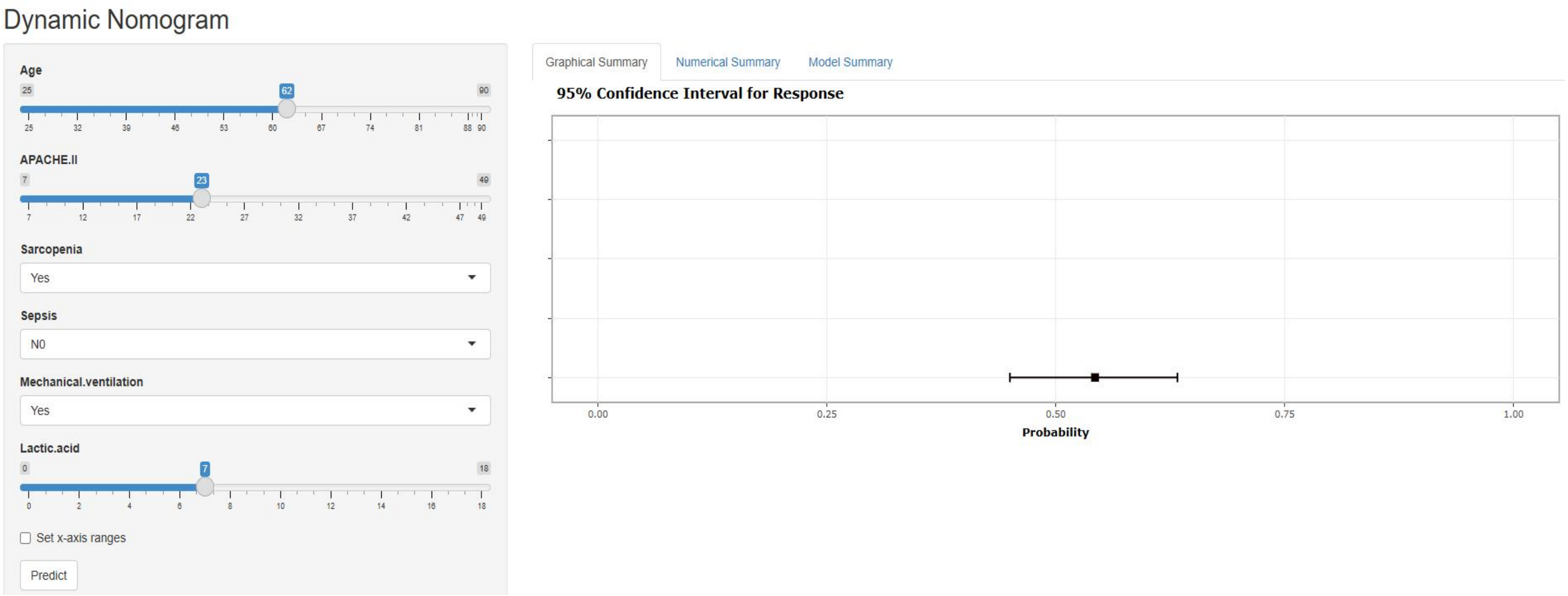

Supplementary Figure 9
Online dynamic nomogram for ICU-AW risk prediction.
